# The Role of the Environment (Water, Air, Soil) in the Emergence and Dissemination of Antimicrobial Resistance: A One Health Perspective

**DOI:** 10.3390/antibiotics14080764

**Published:** 2025-07-29

**Authors:** Asma Sassi, Nosiba S. Basher, Hassina Kirat, Sameh Meradji, Nasir Adam Ibrahim, Takfarinas Idres, Abdelaziz Touati

**Affiliations:** 1 Laboratory of Microbiology and Molecular Biology, Departement of Biochemistry, Faculty of Sciences, Badji Mokhtar University, Annaba 23000, Algeria; asma.sassi@univ-annaba.dz; 2 Department of Biology, College of Science, Imam Mohammad Ibn Saud Islamic University (IMSIU), Riyadh 13318, Saudi Arabia; nsbasher@imamu.edu.sa (N.S.B.); naabdalneim@imamu.edu.sa (N.A.I.); 3 Laboratoire de Recherche des Interactions, Biodiversité, Écosystèmes et Biotechnologie, Faculté des Sciences, Département des Sciences de la Nature et de la Vie, Université 20 Aout 1955-Skikda, Skikda 21000, Algeria; hasnakrt17@gmail.com; 4 Faculty of Nature and Life Sciences, Mohamed-Cherif Messaadia University, Souk Ahras 41000, Algeria; s.meradji@univ-soukahras.dz; 5 Laboratory for Livestock Animal Production and Health Research, Rabie Bouchama National Veterinary School of Algiers, Issad ABBAS Street, BP 161 Oued Smar, Algiers 16059, Algeria; 6 Laboratoire d’Ecologie Microbienne, Faculté des Sciences de la Nature et de la Vie, Université de Bejaia, Bejaia 06000, Algeria; abdelaziz.touati@univ-bejaia.dz

**Keywords:** antimicrobial resistance, antibiotic-resistant bacteria, environmental pollution, One Health, horizontal gene transfer, metagenomics, waterborne pathogens

## Abstract

Antimicrobial resistance (AMR) has emerged as a planetary health emergency, driven not only by the clinical misuse of antibiotics but also by diverse environmental dissemination pathways. This review critically examines the role of environmental compartments—water, soil, and air—as dynamic reservoirs and transmission routes for antibiotic-resistant bacteria (ARB) and resistance genes (ARGs). Recent metagenomic, epidemiological, and mechanistic evidence demonstrates that anthropogenic pressures—including pharmaceutical effluents, agricultural runoff, untreated sewage, and airborne emissions—amplify resistance evolution and interspecies gene transfer via horizontal gene transfer mechanisms, biofilms, and mobile genetic elements. Importantly, it is not only highly polluted rivers such as the Ganges that contribute to the spread of AMR; even low concentrations of antibiotics and their metabolites, formed during or after treatment, can significantly promote the selection and dissemination of resistance. Environmental hotspots such as European agricultural soils and airborne particulate zones near wastewater treatment plants further illustrate the complexity and global scope of pollution-driven AMR. The synergistic roles of co-selective agents, including heavy metals, disinfectants, and microplastics, are highlighted for their impact in exacerbating resistance gene propagation across ecological and geographical boundaries. The efficacy and limitations of current mitigation strategies, including advanced wastewater treatments, thermophilic composting, biosensor-based surveillance, and emerging regulatory frameworks, are evaluated. By integrating a One Health perspective, this review underscores the imperative of including environmental considerations in global AMR containment policies and proposes a multidisciplinary roadmap to mitigate resistance spread across interconnected human, animal, and environmental domains.

## 1. Introduction

Antimicrobial resistance (AMR) has become one of the most pressing global health challenges of the 21st century. It undermines the effectiveness of antibiotics, threatens medical advancements, and contributes to increased mortality, morbidity, and healthcare costs worldwide. The World Health Organization (WHO) has designated AMR as a top public health priority, advocating for a coordinated “One Health” approach that integrates human, animal, and environmental health efforts [1].

**Figure 1:**
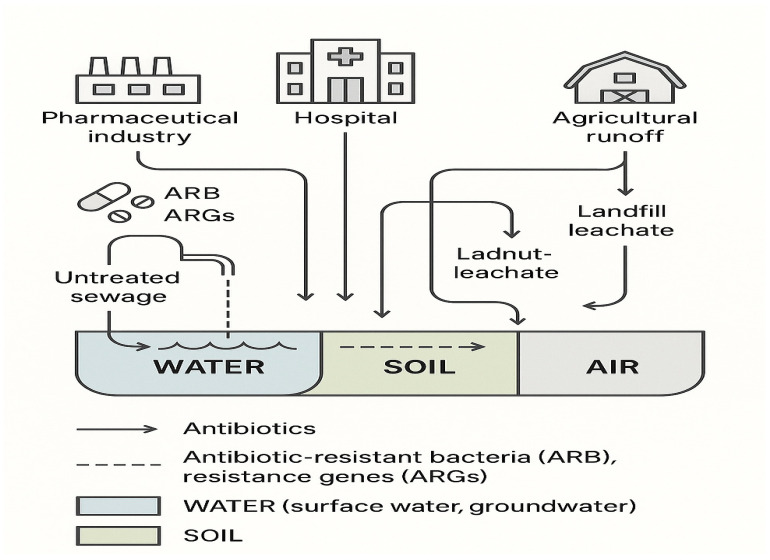
Main entry routes of antibiotics into the environment and their contribution to antimicrobial resistance. Adapted from (Berendonk et al., 2015) [2].

Although AMR research and surveillance have historically centered on clinical settings, the environmental dimension of resistance is gaining increasing attention. Multiple environmental compartments act as reservoirs and conduits for the spread of antibiotic-resistant bacteria (ARB) and antibiotic resistance genes (ARGs). For instance, aquatic environments polluted by untreated or inadequately treated sewage and pharmaceutical effluents [2], agricultural soils exposed to manure or irrigation runoff containing antimicrobial residues [3], and aerosols originating from livestock operations or wastewater treatment facilities [4] all contribute to the dissemination of resistance determinants. These environmental sources not only harbor resistance elements but also enable their transfer across microbial species and ecosystems through horizontal gene transfer [5]. Figure 1 presents an overview of key environmental pathways involved in the emergence and dissemination of resistance.

Addressing the environmental drivers of AMR is essential, particularly in regions lacking stringent waste disposal regulations and environmental safeguards [6,7]. Unlike the more controlled conditions of clinical settings, environmental matrices are highly heterogeneous and often less monitored, allowing resistance to persist and evolve unnoticed.

This review provides a critical synthesis of the role of environmental compartments—namely, water, soil, and air—as reservoirs and transmission routes for AMR. It highlights recent research findings related to the distribution and dynamics of resistance in these environments, drawing from diverse methodological approaches, including environmental surveillance, molecular analyses, and case studies from different global contexts. Special attention is paid to pollution-driven AMR hotspots and the challenges they pose for containment. In doing so, this review emphasizes the importance of integrating environmental perspectives into AMR monitoring, prevention, and mitigation strategies on a global scale.

## 2. Environmental Pathways of Antimicrobial Resistance Transmission

The dissemination of AMR occurs through intricate environmental pathways, enabling ARB and ARGs to persist and spread across diverse ecosystems [8]. These transmission routes encompass aquatic systems, terrestrial reservoirs, and atmospheric dispersion, each playing a pivotal role in the global AMR crisis [2]. The interactions among these pathways exacerbate the risks, fostering the exchange of resistance determinants across environmental, agricultural, and clinical settings.

### 2.1. Aquatic Ecosystems: Critical Reservoirs and Transmission Pathways for Antimicrobial Resistance

Aquatic environments serve as reservoirs and conduits for AMR, facilitating the persistence and horizontal transfer of ARBs and ARGs [9]. The confluence of urban wastewater, agricultural runoff, and industrial effluents creates a selective pressure that enhances the propagation of resistance determinants in aquatic ecosystems [10]. Even advanced wastewater treatment facilities struggle to eliminate mobile genetic elements encoding resistance, further amplifying the environmental burden of AMR [11].

#### 2.1.1. Key Sources of AMR Contamination in Aquatic Systems

**Figure 2:**
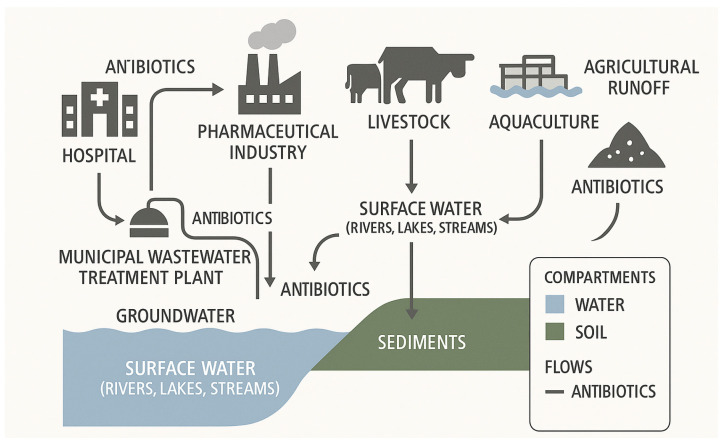
Schematic diagram illustrating the main entry pathways of antibiotics into aquatic environments. Adapted from Maghsodian et al., 2022 [25].

Municipal wastewater treatment plants are significant contributors to the environmental spread of AMR, as they receive high loads of antibiotic residues and resistant bacteria from human excretion [12]. These effluent samples often contain clinically relevant resistance genes such as *bla_CTX-M-15_*, associated with extended-spectrum β-lactamase (ESBL) production, and *mcr-1*, which confers resistance to colistin. Studies have shown that these resistance genes are present in over 80% of effluent samples worldwide, reflecting the pervasive nature of AMR in urban environments [13]. Despite implementing advanced treatment processes, residual antibiotics and mobile genetic elements (MGEs) persist in the treated effluents. These compounds continue circulating through surface waters, facilitating the spread and persistence of resistant pathogens [14]. The presence of these pollutants in the environment underscores the challenge of eliminating AMR from wastewater systems. It highlights the potential for these waters to act as reservoirs and conduits for resistance, ultimately impacting both human and environmental health. The principal entry pathways of antibiotics and resistance genes into aquatic environments are illustrated in Figure 2.

The agricultural sector also plays a crucial role in the dissemination of AMR, particularly through the use of antibiotics in livestock farming. These veterinary antibiotics, such as tetracyclines and macrolides, are commonly administered in food animal production to promote growth and prevent disease [15]. However, the widespread use of these substances has led to the accumulation of resistance determinants in soils, which can leach into nearby water bodies, further exacerbating the issue [16]. Rivers and lakes receiving agricultural runoff have been found to contain significant concentrations of these antibiotics, which in turn drive the selection of resistant pathogens. Notably, strains of *Escherichia coli* ST131, a widely recognized multidrug-resistant pathogen, as well as methicillin-resistant *Staphylococcus aureus* (MRSA), have been isolated from such environments [17,18]. These findings illustrate how agricultural runoff acts as a vector for AMR, contributing to the emergence and spread of resistant organisms that can impact human health and the broader ecosystem.

Pharmaceutical effluents represent another critical source of AMR in the environment, particularly in regions where pharmaceutical manufacturing is concentrated. Wastewater discharged from pharmaceutical manufacturing plants has been identified as a significant hotspot for the selection of AMR [19]. Research conducted in industrial hubs such as Hyderabad, India, has revealed alarmingly high ciprofloxacin concentrations exceeding 1 mg/L, 30,000 times higher than the minimum selective concentration required for resistance development [20]. Similar findings have been reported worldwide. For example, in China, pharmaceutical effluents have reached ciprofloxacin concentrations up to 31 mg/L, and surface waters near manufacturing sites have shown levels as high as 2.5 mg/L [21]. In the United States, elevated concentrations of antibiotics such as ciprofloxacin, azithromycin, and sulfamethoxazole have been detected in surface waters and municipal effluents, especially in the Midwest and Northeast regions [22]. In Europe, a comprehensive survey of the Danube River revealed the presence of multiple antibiotic-resistant bacteria and high levels of pharmaceutical residues along its 2300 km course [22]. A recent global survey found that ciprofloxacin concentrations in rivers exceeded safe levels in 64 countries, with notable hotspots not only in India but also in China, Nigeria, Pakistan, and several European countries [23]. These examples underscore that pharmaceutical pollution and its contribution to environmental antibiotic resistance areglobal phenomena, not limited to any single country or region. These elevated concentrations promote the emergence of hypermutagenic strains, such as *Pseudomonas aeruginosa*, which can rapidly acquire mutations and adapt to the selective pressure exerted by the antibiotics [24]. Such high concentrations of antibiotics in effluents underscore the need for stricter regulations and more effective waste management strategies in pharmaceutical industries to mitigate their contribution to environmental AMR. These industrial discharges are a significant source of AMR contamination, emphasizing the need for comprehensive approaches to address pharmaceutical waste and limit its environmental impact.

#### 2.1.2. Mechanisms of Resistance Propagation in Aquatic Systems

**Figure 3:**
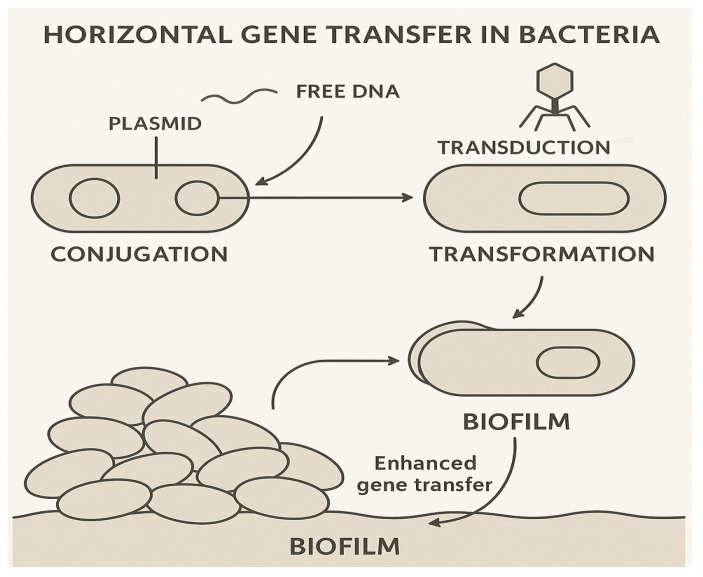
Schematic diagram illustrating the main mechanisms of horizontal gene transfer (HGT) in bacteria: conjugation, transformation, and transduction. Adapted from Kunhikannan et al., 2021 [34].

Horizontal gene transfer (HGT) plays a pivotal role in the spread of AMR in aquatic environments, particularly in biofilms. These biofilms offer a conducive microenvironment for conjugating resistance genes between environmental and pathogenic bacteria, enhancing the horizontal transmission of resistance traits [26,27]. Studies have highlighted the high-frequency transfer of carbapenem resistance genes, such as *bla_NDM-1_*, in this context, within wastewater biofilms. Notably, such genes have been transferred on Incx3 plasmids, with conjugation rates reaching as high as 10^2^ transconjugants per donor cell, underscoring gene transfer efficiency in these aquatic settings [28,29]. This phenomenon is of significant concern, as it facilitates the rapid dissemination of resistance genes across bacterial populations, exacerbating the global threat of AMR. The main mechanisms of horizontal gene transfer in aquatic environments are illustrated in Figure 3.

In addition to HGT, sub-inhibitory concentrations of antibiotics present in aquatic environments can exert selection pressure on bacterial populations, even at levels as low as nanograms to micrograms per liter. Such concentrations, often resulting from residual antibiotics from human and agricultural sources, are sufficient to trigger bacterial stress responses, notably the SOS response [30]. This stress response, in turn, leads to an increase in integron activity, which enhances the acquisition of resistance genes [31]. Among the genes acquired under these conditions are *sul1*, which confers resistance to sulfonamides, and *qnrS*, which is associated with quinolone resistance [32]. The presence of sub-inhibitory antibiotics thus acts as a continuous, albeit often unnoticed, driver of resistance gene accumulation, further complicating efforts to combat bacterial resistance.

Furthermore, the persistence of extracellular DNA in the environment significantly contributes to the spread of AMR. MGEs, such as the *tet*(W) gene, can remain stable in aquatic sediments for extended periods, often over 40 days, due to their protection by organic matter and clay particles [33]. This persistence allows the transformation of environmental pathogens, such as *Vibrio cholerae*, with resistance genes that may have been previously acquired by other bacteria in the surrounding environment [33]. The ability of resistance genes to survive outside of bacterial cells and remain viable in the environment further complicates the mitigation of AMR, as it creates a reservoir of genetic material that can potentially be taken up by various pathogens, thereby enhancing the spread of resistance across different bacterial species.

#### 2.1.3. Challenges in Wastewater Treatment and Mitigation Strategies

Despite significant technological advancements in wastewater treatment processes, WWTPs remain insufficient in eliminating antibiotic residues and AMR determinants from treated effluents. Conventional methods such as ultraviolet (UV) disinfection, which aim to reduce bacterial loads in wastewater, have shown limited success in degrading extracellular DNA that carries antibiotic resistance genes, such as *ermB*, which is associated with macrolide resistance [35]. This failure to eliminate MGEs means that resistance genes persist in the treated water, allowing resistant bacteria to continue circulating in the environment and potentially leading to the spread of AMR across ecosystems [36].

Further complicating the issue, chlorine disinfection, a widely used method for pathogen control in wastewater, has been observed to select chlorine-resistant strains of *Enterococcus faecium* that harbor *vanA*, a gene responsible for vancomycin resistance [37]. These chlorine-resistant strains can recolonize distribution networks within a mere 72 h after treatment, undermining the effectiveness of disinfection processes and contributing to the persistence and spread of resistance in the environment [38]. This phenomenon underscores the limitations of conventional disinfection techniques in addressing the complexity of AMR in wastewater.

Additionally, recycling biosolids from wastewater treatment, often employed as a means of agricultural fertilization, poses further challenges in combating AMR. The anaerobic digestion of biosolids can enrich spore-forming bacteria, such as *Clostridioides difficile*, which carry resistance determinants like *cfr*, a gene conferring resistance to linezolid [39,40]. When these biosolids are applied to agricultural fields, they can reintroduce AMR into terrestrial ecosystems, thus perpetuating the cycle of resistance. This process highlights a significant environmental pathway through which resistant bacteria and resistance genes are disseminated into the broader ecosystem, exacerbating the global challenge of AMR [14]. Therefore, while advancements in wastewater treatment technologies have made progress in reducing bacterial contamination, the persistence of antibiotic residues, resistance genes, and resistant bacteria in treated effluents and biosolids continues to be a significant concern in the fight against AMR. The main water and wastewater treatment technologies, their advantages, limitations, and potential contribution to AMR are summarized in Table 1.

### 2.2. Terrestrial Reservoirs: Soil as a Dynamic Reservoir for Antimicrobial Resistance Evolution

Soil ecosystems are not mere passive repositories of ARBs and ARGs but active bioreactors where resistance determinants evolve, recombine, and disseminate. The interplay of agricultural practices, microbial ecology, and anthropogenic pressures transforms soil into a nexus for AMR amplification, with implications spanning environmental and clinical settings [48].

#### 2.2.1. Drivers of ARG Accumulation in Soil

Livestock manure has become a significant vector for introducing antibiotics into agricultural soils, with studies showing that it accounts for approximately 30–90% of the antibiotics administered to animals, including commonly used compounds such as tetracyclines and sulfonamides [49]. When manure is applied to soils, it creates sub-inhibitory concentrations of these antibiotics, typically ranging from 0.1 to 100 µg/kg, which fosters the selective pressure needed to proliferate antibiotic-resistant microorganisms [50,51]. This resistance is not a transient phenomenon, as a single manure application has been reported to deposit between 10^6^ and 10^8^ copies of ARGs per gram of soil, including genes conferring resistance to tetracycline (*tet*(M)) and macrolides (*erm*(B)) [52]. These ARGs are deposited and integrate stably into the soil’s microbial communities, where they can persist for over 20 years, further embedding resistance into the agricultural ecosystem. This long-term persistence raises substantial concerns about the continued presence of these resistance markers in the environment, potentially leading to long-lasting ecological consequences.

In addition to the persistence of resistant genes, research has demonstrated that manure from swine, in particular, significantly enhances the population of *Clostridioides difficile* spores in agricultural soils [53]. This bacterium, which is known to harbor resistance genes such as cfr (linezolid resistance) and *vanA* (vancomycin resistance), can increase by up to 100-fold following manure amendments [54]. The presence of these resistant strains in the soil increases the risk of environmental contamination and poses serious zoonotic risks. These resistant strains can enter the food chain through soil contamination or contact humans and animals directly, amplifying the spread of resistance across various ecological niches.

The use of treated wastewater for irrigation compounds these issues by introducing not only antibiotic residues but also heavy metals like copper and zinc, as well as chemical disinfectants. These substances exert co-selection pressures, where organisms resistant to one agent may also develop resistance to others, particularly when they share similar resistance mechanisms [55].

Heavy metals such as copper and zinc are well-documented co-selective agents for antimicrobial resistance. Their presence in soil and water can select for bacteria carrying both metal resistance genes and antibiotic resistance genes, often located on the same mobile genetic elements (co-resistance), or via shared efflux pumps (cross-resistance) [56,57,58,59]. For instance, the czcA gene confers resistance to zinc and cadmium, and is frequently found in proximity to sul1, a sulfonamide resistance gene, on integrons or plasmids [58]. Experimental studies have shown that exposure to copper at environmentally relevant concentrations (10–100 mg/L) rapidly increases the abundance of both metal and antibiotic resistance genes in bacterial communities, even in the absence of antibiotics [56,58,59]. Similarly, pre-exposure to zinc has been shown to accelerate the development of ciprofloxacin resistance in *E. coli* [57].

Chemical disinfectants, such as quaternary ammonium compounds (QACs), triclosan, and chlorhexidine, are also linked to the selection and persistence of antibiotic resistance [60,61]. Overuse or improper use of these disinfectants can induce genetic adaptations in bacteria—such as mutations in efflux pump genes (e.g., qacA/B, marA, mexA-mexB-oprM)—that confer decreased susceptibility to both biocides and multiple antibiotics [60,61]. This phenomenon, known as cross-resistance, has been documented in clinically relevant strains of *E. coli*, *P. aeruginosa*, and *S. aureus* [61].

High concentrations of resistance genes such as czcA, which confers resistance to zinc and cadmium, and sul1, which provides resistance to sulfonamides, have been found abundant in soils irrigated with treated wastewater. This co-selection process is especially concerning because it can spread multidrug-resistant organisms within agricultural systems, further complicating efforts to manage and mitigate antimicrobial resistance in the environment and human health [58,60,61].

#### 2.2.2. Soil as a Hotspot for HGT

**Figure 4:**
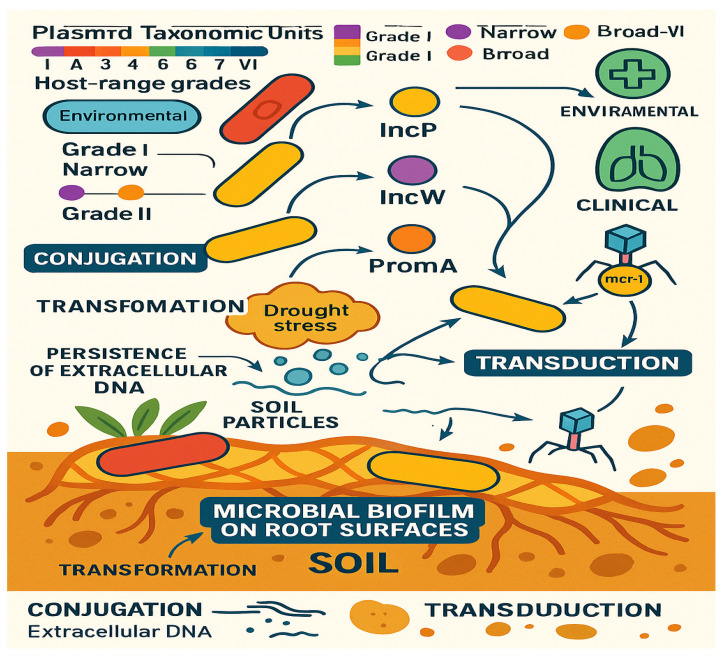
Schematic diagram illustrating HGT in soil environments, including plasmid host-range diversity, biofilm-mediated plasmid exchange, transformation (with extracellular DNA binding to soil particles), and transduction via bacteriophages. Adapted from Ciach et al., 2024 [66]; Liu et al., 2024 [67]; Aminov, 2011 [68]; Li et al., 2023 [69].

Recent large-scale genomic analyses have revealed the central role of plasmids in facilitating HGT across diverse bacterial taxa and ecological niches. A global study analyzing over 10,000 plasmid genomes uncovered a complex network of plasmid taxonomic units (PTUs), highlighting the evolutionary pathways genes, including those conferring AMR—can disseminate broadly [62]. These PTUs were classified into six host-range grades, from narrow host plasmids (Grade I) to broad-host-range plasmids (Grade VI), the latter capable of moving between bacteria of different phyla. Over 60% of the plasmids examined were found to transcend species boundaries, underlining their role as vectors of gene exchange across taxonomic and ecological barriers. Broad-host-range plasmids such as IncP, IncW, and PromA groups were identified as key mediators of gene flow between environmental and clinical bacterial populations. This capacity enables the rapid spread of AMR genes from ecological reservoirs, such as soil or water, to human pathogens, emphasizing the need to understand plasmid dynamics in the context of One Health [62]. This demonstrates the potential for clinically relevant resistance traits to emerge in environmental reservoirs, with soil as a key medium for exchanging resistance determinants between environmental and pathogenic bacteria. Additionally, prevalent on root surfaces, microbial biofilms further enhance the capacity for plasmid exchange [63]. This interaction highlights the importance of biofilm-mediated gene transfer in facilitating the spread of resistance genes, even in environments that are not directly exposed to antibiotics. The main mechanisms and ecological networks of horizontal gene transfer in soil environments are illustrated in Figure 4.

In addition to conjugation, other mechanisms of HGT, such as transformation and transduction, also play significant roles in the movement of AMR genes within soil ecosystems. Transformation occurs when extracellular DNA carrying resistance genes, such as *sul2* and *intI1* (which encode sulfonamide resistance and class 1 integrons, respectively), binds to soil particles like clay and remains in a viable form for extended periods, often exceeding a year, even under arid conditions [64]. This persistence of extracellular DNA ensures that soil remains a reservoir of transferable genetic material, capable of being incorporated into other bacterial cells under the right conditions. Transduction, another key gene transfer mechanism, is mediated by bacteriophages that carry genetic material between bacterial species. In soil environments, bacteriophages facilitate the transduction of the *mcr-1* gene, which confers resistance to colistin, between *Enterobacteriaceae* and environmental *Aeromonas* species. This process is particularly pronounced under drought stress, where the frequency of transduction events is elevated, thereby enhancing the spread of resistance genes within microbial communities [65]. These findings emphasize the complex and dynamic nature of HGT in soil ecosystems, where various mechanisms interact to promote the persistence and dissemination of antimicrobial resistance.

#### 2.2.3. Metagenomic Insights into Soil Resistomes

Recent metagenomic studies have revealed significant concerns regarding the complexity and potential risks associated with soil resistomes [70,71]. These studies highlight the considerable diversity of ARGs in agricultural soils, which has become a growing environmental issue. Agricultural soils harbor a range of 200 to 500 distinct ARG subtypes, some clinically significant. Among these, variants such as *vanA* and *bla_KPC_* stand out because they confer resistance to critical antibiotics like glycopeptides and carbapenems, which are used to treat various severe infections [72]. The widespread presence of these genes in soil poses a direct threat to human health, as it may contribute to the environmental dissemination of resistance traits that could eventually affect clinical treatment outcomes.

In addition to the sheer diversity of ARGs, cross-kingdom gene transfer has emerged as another alarming trend. Soil fungi, particularly species from the *Aspergillus genus*, have been identified as unexpected reservoirs for bacterial ARGs. A noteworthy example is the *ampC* gene, which confers resistance to β-lactam antibiotics. The detection of ampC within fungal mitochondrial genomes provides strong evidence of horizontal gene transfer between bacterial and fungal communities in the soil [66,73]. This finding challenges traditional notions about gene transfer and is supported by multiple genomic and metagenomic studies that have identified bacterial antibiotic resistance genes within fungal genomes [66,74,75,76]. It underscores the need for further investigation into the intricate microbial interactions that may facilitate the spread of resistance genes across different domains of life. The ability of fungi to harbor bacterial resistance genes adds a new layer of complexity to the dynamics of ARG spread in the environment, highlighting the importance of considering all microbial communities when assessing the spread of antimicrobial resistance [66,75,76,77].

Furthermore, the geographic distribution of ARGs in soil varies significantly depending on farming practices, providing additional insight into the relationship between agricultural activity and the persistence of resistance genes. In Europe, soils located near intensive poultry farms have been found to contain up to ten times higher levels of erm(B), a gene associated with resistance to macrolides, compared to soils from organic farming systems [78,79,80]. This stark contrast can be directly linked to the extensive use of veterinary antibiotics in conventional farming systems, which are widely used to prevent and treat infections in livestock. Studies have shown that such antibiotic use correlates with increased abundance and persistence of ARGs in soil [51,81,82]. The higher concentration of ARGs in soils from intensive farming systems suggests that antibiotic use in agriculture is critical in fostering the selection and persistence of resistant strains in the environment [83]. Such findings highlight the significant influence of agricultural practices on the spread of antimicrobial resistance and emphasize the need for sustainable farming practices that reduce the reliance on antibiotics.

#### 2.2.4. Atmospheric Transport Dynamics

ARBs exhibit significant resilience and adaptability, enabling their persistence in various environmental conditions. These bacteria can survive in the air for extended periods, ranging from 8 to 72 h, thanks to protective organic coatings, such as lipids derived from manure. These coatings protect the bacteria from harmful ultraviolet (UV) radiation and desiccation, which would otherwise impair their viability [84]. This ability to withstand harsh environmental conditions contributes to the widespread dispersal of ARBs across different ecosystems.

The spread of ARBs, including MRSA, is facilitated by wind-driven processes, further enhancing their transmission on local and continental scales. On a regional scale, studies have shown that MRSA strains, such as MRSA-CC398, originating from pig farms in the Netherlands, can be detected in 30% of residential areas within a 3 km radius of the farms [85]. This finding underscores the significant role of airborne dissemination in propagating resistant strains from animal reservoirs to human populations. On a larger, continental scale, wind-borne particulate matter can transport resistance genes across vast distances. For example, dust storms originating from North African feedlots have been shown to carry the *bla_CTX-M_* genes responsible for ESBL production to Southern Europe. These airborne particles contribute to the environmental burden of antimicrobial resistance, with deposition rates of up to 10^7^ ARG copies per square meter per day [86].

In addition to the direct transmission of resistant bacteria, particulate matter, suchas PM_2.5_ and PM_10_, serves as a vector for genetic material that facilitates the spread of resistance [87]. These fine particles, less than 10 μm in diameter fine particles can carry conjugative plasmids, such as IncHI2, which harbor resistance genes like *mcr-1*, responsible for colistin resistance. The adsorption of these plasmids onto silica surfaces further enhances their stability and mobility in the atmosphere [88]. Moreover, studies have detected extracellular DNA encoding *vanA*, a gene responsible for vancomycin resistance, in urban air samples, highlighting the widespread distribution of genetic material that promotes resistance [89].

**Figure 5:**
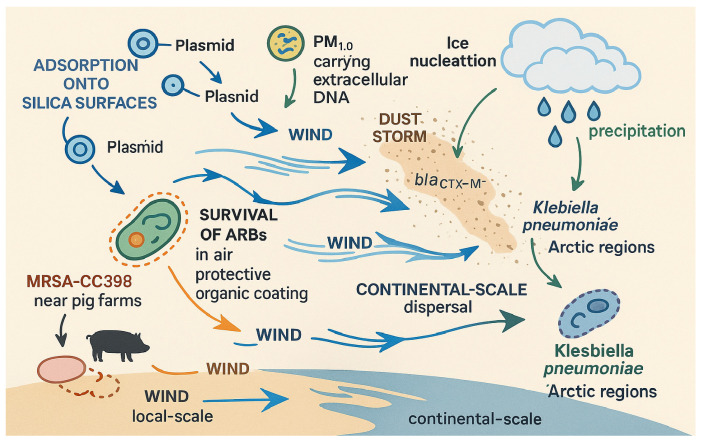
Schematic diagram illustrating the main mechanisms and pathways of atmospheric transport and dispersal of antimicrobial-resistant bacteria (ARBs) and resistance genes, including survival in aerosols, wind-driven dispersal, association with particulate matter, and long-range transport via ice nucleation and precipitation. Adapted from Zhou et al., 2023 [87].

Interestingly, particulate matter can also facilitate the long-range dispersal of ARBs through a process known as ice nucleation. Fine particles laden with ARBs can act as nuclei, promoting cloud formation and enabling bacteria to be transported across intercontinental distances via precipitation. For instance, *Klebsiella pneumoniae*, a pathogen associated with antimicrobial resistance, has been recovered from rainfall in pristine Arctic regions, illustrating how airborne bacteria can be carried over vast distances and deposited in remote locations [90]. This highlights the global scale of the problem and the complex mechanisms by which ARBs can spread, potentially impacting regions far removed from their sources. The main mechanisms and pathways of atmospheric transport and dispersal of antimicrobial-resistant bacteria and genes are illustrated in Figure 5.

#### 2.2.5. Horizontal Gene Transfer in Air

Emerging evidence increasingly supports the idea that airborne conditions play a significant role in facilitating genetic exchange among bacterial populations, particularly in AMR. Several studies have shown that mechanisms of HGT, such as conjugation and transduction, can occur in airborne particles, contributing to the spread of resistance genes across vast distances [91,92].

One of the primary mechanisms facilitating HGT in the air is conjugation, a process where genetic material is transferred between bacterial cells through direct contact. Studies have shown that *Acinetobacter* species, known for their role in transmitting resistance genes, can transfer plasmids encoding carbapenem resistance, such as the *bla_NDM-1_* gene, within bioaerosols [93,94]. For instance, Scoffone et al. demonstrated that *Acinetobacter baumannii*, a significant pathogen in hospital-associated infections, can transfer *bla_NDM-1_* plasmids within bioaerosols. This highlights the potential for spreading antibiotic resistance through the atmosphere, especially as wind-borne bioaerosols can carry resistant bacteria over long distances [95].

**Figure 6:**
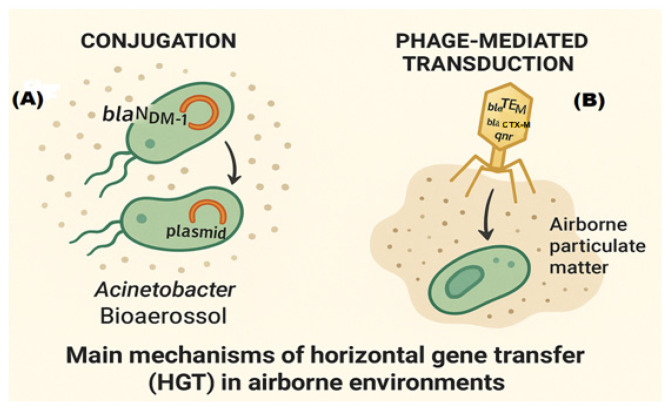
Schematic diagram illustrating the main mechanisms of horizontal gene transfer (HGT) in airborne environments: (**A**) Conjugation between bacteria within bioaerosols, exemplified by *Acinetobacter* species transferring *blaNDM-1* plasmids; (**B**) phage-mediated transduction, where bacteriophages carry and transfer antibiotic resistance genes (ARGs) such as *blaTEM*, *blaCTX-M*, and qnr within airborne particulate matter. Adapted from Colomer-Lluch et al., 2011 [96]; Ye et al., 2021 [97].

In addition to conjugation, phage-mediated transduction is another crucial mechanism of genetic exchange in airborne and environmental conditions. Bacteriophages, viruses that infect bacteria, are abundant in various environments and have been detected in the DNA fraction of particulate matter. These phages can carry and transfer ARGs, including those conferring resistance to critical antibiotics. The presence of ARGs, such as *blaTEM*, *blaCTX-M*, and qnr, in the bacteriophage DNA fraction obtained from diverse environmental samples—including wastewater and river water—has been demonstrated. This finding highlights the role of phages as vectors for horizontal gene transfer in natural environments, potentially including aerosols, thereby contributing to the dissemination of resistance genes. The capacity of phages to mediate gene transfer enhances bacterial genetic diversity and represents a significant route for the spread of antimicrobial resistance across different ecosystems [96]. The main mechanisms of horizontal gene transfer in airborne environments, including conjugation and phage-mediated transduction, are illustrated in Figure 6.

## 3. Impact of Environmental Pollution on AMR

Environmental pollution is a global catalyst for the evolution and dissemination of antimicrobial resistance (AMR), creating selective pressures that favor resistant bacteria and facilitating the horizontal transfer of resistance genes across all ecosystems [98]. The main environmental drivers include pharmaceutical and industrial waste, untreated sewage, agricultural runoff, and air pollution, each contributing uniquely to the selection, persistence, and spread of AMR on every continent [98,99,100,101,102].

### 3.1. Key Pollutants, Sources, Concentrations, and Global Locations

#### 3.1.1. Pharmaceutical Waste

Pharmaceutical residues are detected worldwide, from rivers in Europe and North America to major rivers in Asia and Africa. For example, a global survey found antibiotics in rivers in over 100 countries, with concentrations of ciprofloxacin reaching up to 31 mg/L in pharmaceutical effluents in India and 1.2 mg/L in the Danube River, Europe [99,100]. In China, concentrations of sulfonamides in surface waters near manufacturing hubs can reach 2.5 mg/L [101]. These sub-inhibitory concentrations (10–100 ng/L or higher) select for resistance genes such as *blaCTX-M*, *qnrS*, and *mcr-1* in environmental bacteria [99,101].

#### 3.1.2. Wastewater (Treated and Untreated)

Both untreated and inadequately treated municipal wastewater are major sources of ARB and ARGs globally, especially in low- and middle-income countries [98,100]. Urban sewage in 60 countries has revealed a clear geographic distinction in AMR gene abundance, with the highest levels in Asia, Africa, and South America [100]. Wastewater—whether treated or not—releases high loads of carbapenem-resistant Enterobacteriaceae (CRE), vanA-positive *Enterococcus*, and integrons (intI1) into aquatic environments, with ARG concentrations up to 10^8^ copies/mL [19]. Discharges can impact both coastal and inland waters.

#### 3.1.3. Agricultural Runoff

Intensive agriculture is a major driver of AMR, with conventionally farmed soils harboring more ARGs than organic systems [98,101]. For example, soils near intensive poultry farms in Europe contain up to ten times higher levels of erm(B) than organic farms [101]. In the US and China, tetracycline and sulfonamide resistance genes persist in soils for decades after manure application [98,101]. Heavy metals like copper and zinc, commonly found in fertilizers and animal feed, co-select for ARGs such as czcA and sul1 [98,101].

#### 3.1.4. Industrial Waste and Microplastics

Industrial discharges, including from textile manufacturing and microplastics, are global issues. In estuaries of the UK and China, industrial legacy pollution correlates with high levels of sul1, ermB, and marA genes [19,98]. Microplastics sampled from rivers in Europe, Asia, and Africa carry ARGs and promote biofilm formation by resistant bacteria [19,98].

#### 3.1.5. Air Pollution

Recent studies show that air pollution (PM2.5 and PM10) can carry ARGs and resistant bacteria across continents [19].

For example, dust storms from North Africa have transported *blaCTX-M* genes to Southern Europe, while ARGs have been detected in urban air samples in China, Europe, and the US [19]. The main environmental pollutants contributing to antimicrobial resistance, their major sources, typical concentrations, and global locations are summarized in Table 2.

### 3.2. Synergistic Effects of Combined Pollutants

While the individual roles of antibiotics, metals, and microplastics in promoting AMR have been discussed above, recent research emphasizes that their combined presence in the environment can have synergistic effects that go beyond the sum of their individual impacts. These interactions can lead to enhanced selection, persistence, and horizontal transfer of resistance genes, representing an emerging concern for environmental and public health.

For example, co-contamination with metals and antibiotics can significantly amplify resistance gene expression. A recent study demonstrated that a mixture of zinc (10 mg/L) and oxytetracycline (0.1 mg/L) resulted in a 100-fold increase in tetA gene expression in *Bacillus* species from soil, compared to exposure to either contaminant alone [106]. This finding suggests that metal-antibiotic mixtures may accelerate the evolution and dissemination of resistance traits in contaminated soils.

Microplastics act as both vectors and catalysts for gene transfer. Unlike previous sections that focused on their role as passive carriers, new evidence shows that microplastics such as 1 µm polystyrene particles can actively enhance the horizontal transfer of resistance genes. Specifically, the presence of these particles increased the transfer rate of the *blaTEM* gene by 50% between *Vibrio* species in marine environments [107]. This highlights the unique role of microplastics in creating microenvironments that facilitate gene exchange among bacteria.

Overall, these findings underscore the importance of considering pollutant mixtures and their interactions, rather than assessing each contaminant in isolation, when evaluating environmental drivers of AMR. Future mitigation strategies should account for these synergistic effects to more effectively curb the spread of resistance [108].

### 3.3. Quantitative Evidence of Pollution—AMR Links

The presence and proliferation of AMR genes in environmental reservoirs—such as water, soil, and air—pose a growing threat to public health. Among these, waterborne resistance is particularly concerning. Studies have shown that even low concentrations of antibiotics, such as ciprofloxacin, can exert a selective pressure on microbial communities in aquatic ecosystems.

For instance, Kraupner et al. demonstrated that ciprofloxacin concentrations as low as 1 μg/L significantly altered the composition of complex aquatic bacterial biofilms. This exposure led to the enrichment of mobile quinolone resistance genes, especially *qnrS*, emphasizing that even minimal levels of pharmaceutical contaminants can contribute to the selection and persistence of resistance determinants in natural environments [109]. Similarly, Li et al. (2016) found that in soil amended with manure, lower ciprofloxacin concentrations (0.04–0.4 mg/kg) slowed the dissipation of plasmid-mediated quinolone resistance (PMQR) genes, including *qnrS*, and promoted the survival of resistant bacterial taxa [110].

Similarly, in agricultural soils, manure has been shown to contribute to a significant rise in the levels of sul1, a sulfonamide resistance gene. Within just one year of manure application, sul1 levels in the soil can increase from 10^3^ to 10^6^ copies per gram, highlighting agricultural practices’ rapid and sustained influence on the soil resistome [48]. Furthermore, airborne transmission is a significant vector for the spread of AMR, particularly in agricultural settings.Studies have demonstrated that particulate matter (PM2.5) near livestock farms contains substantial concentrations of ARB.For instance, research has identified the presence of airborne MRSA CC398 in pig farms, indicating a potential risk for airborne transmission of resistant pathogens [111].

## 4. Antimicrobial Resistance in the Environment: A Global Perspective

### 4.1. Global Distribution of Environmental AMR Hotspots

The variability in AMR across different regions is influenced by several key drivers that reflect each region’s unique socio-economic and infrastructural contexts. In low- and middle-income countries (LMICs), sanitation gaps represent a major contributor to the spread of ARB. Around 80% of wastewater in these countries is estimated to remain untreated, releasing substantial quantities of ARB into rivers used for drinking and irrigation. This untreated wastewater, with concentrations ranging from 10^6^ to 10^8^ ARB per milliliter, provides an environment for the proliferation and transmission of resistant strains, significantly impacting public health, especially in communities dependent on these water sources for daily consumption and agricultural purposes [112]. In addition to sanitation issues, agricultural pressures in LMICs further exacerbate the problem of AMR. Projections indicate that veterinary antibiotic use, especially in poultry and aquaculture, will rise by 67% by 2030. This increase is expected to enhance the selective pressure on microbial populations, fostering the development and spread of resistant strains in animal and environmental reservoirs [113].

**Figure 7:**
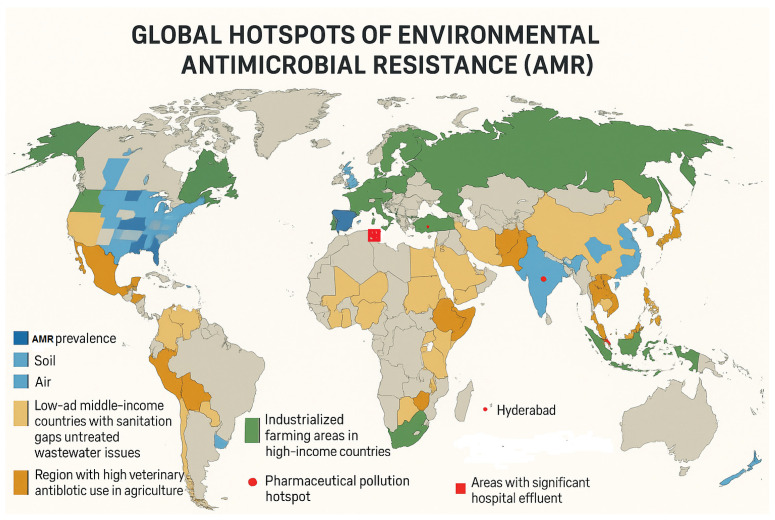
Global hotspots and environmental drivers of antimicrobial resistance (AMR).

Conversely, high-income countries (HICs) face different yet significant challenges related to the persistence and spread of AMR. Industrialized farming practices in HICs contribute substantially to the environmental burden of antimicrobial resistance. Despite regulatory measures, it is estimated that 70% of medically necessary antibiotics in the United States are used in livestock farming, which continues to enrich agricultural soils with resistance genes, particularly erm(B) and tet(M). These genes confer resistance to macrolides and tetracyclines, respectively, and are prevalent in both farm environments and the surrounding ecosystems, where they can be disseminated through manure, runoff, and other agricultural activities [114]. In high-income countries (HICs), hospital effluents are recognized as critical sources of AMR dissemination into the environment. These effluents often contain elevated concentrations of carbapenemase genes, including *bla_KPC_* and *bla_OXA-48_*, which can persist through conventional wastewater treatment processes. A study conducted by Nasri et al. [115]. In Tunisian hospitals, reported concentrations of carbapenemase genes such as *bla_OXA-48_* reaching up to 10^4^ copies per milliliter in untreated hospital wastewater. These genes were detected using quantitative PCR techniques, emphasizing their prevalence and potential for environmental dissemination [115]. The global distribution of environmental AMR hotspots, including regions with high prevalence in soil, air, and water, as well as areas impacted by untreated wastewater, intensive agriculture, and pharmaceutical or hospital effluents, is illustrated in Figure 7.

World map highlighting regions with high AMR prevalence in soil, air, and water; low- and middle-income countries with sanitation gaps and untreated wastewater; regions with intensive veterinary antibiotic use; industrialized farming areas; pharmaceutical pollution hotspots (e.g., Hyderabad); and areas with significant hospital effluence.

### 4.2. Regional Case Studies

The presence of AMR in environmental reservoirs is a growing concern across various regions, with substantial implications for public health. In Sub-Saharan Africa, the sanitation crisis plays a critical role in the spread of AMR. In Nigeria, studies have demonstrated the presence of extended-spectrum beta-lactamase (ESBL)-producing *Escherichia coli*, particularly those carrying the *bla_CTX-M-15_* gene, in drinking water sources.

For example, a study conducted on selected rivers in Ibadan revealed the presence of *bla_CTX-M_* genes in *E. coli* isolates from surface waters, indicating significant environmental contamination and public health risk in communities relying on these water bodies [116].

Additionally, research conducted in Oyo State confirmed the presence of ESBL-producing Gram-negative bacteria, including *E. coli*, in various surface and underground water sources, with a notable prevalence of the *bla_CTX-M_* gene [117].

This widespread contamination highlights the critical intersection between inadequate sanitation infrastructure and AMR proliferation, exacerbating health disparities in low-resource settings. In Ghana, genomic surveillance has revealed the presence of *Klebsiella pneumoniae* strains carrying the *bla_NDM-1_* gene—an enzyme responsible for carbapenem resistance—across hospital, environmental, and animal samples, underscoring the pervasive nature of resistance transmission across One Health domains. These findings raise serious concerns about the ecological dissemination of highly resistant pathogens and their implications for vulnerable populations, including neonates at risk of sepsis-related mortality [118].

In India and Southeast Asia, pharmaceutical pollution and agricultural practices significantly contribute to the environmental load of resistant pathogens. In Hyderabad, India, the pharmaceutical industry discharges effluents containing high concentrations of ciprofloxacin, around 1.5 mg/L, which has been shown to select for *qnrS*—a gene associated with quinolone resistance—in 90% of aquatic *V. cholerae* populations [24]. This exemplifies how pharmaceutical contamination in marine environments can directly impact the microbial resistance profiles of local ecosystems. In Vietnam, the widespread use of oxytetracycline in aquaculture further contributes to AMR. Shrimp farms apply 500 mg/kg of this antibiotic, significantly increasing the prevalence of antibiotic resistance genes. A study by Le and Munekage (2004) [119] observed that antibiotic residues, including tetracyclines, were present in water and sediment samples from shrimp ponds in mangrove areas, raising concerns about these practices’ environmental impact. Although the specific prevalence of the *tetA* gene was not quantified in the study, such residues can foster the development and persistence of resistance in these environments. This, in turn, exacerbates the spread of tetracycline-resistant bacteria, contributing to a growing environmental health risk [119].

In Europe and North America, where regulatory frameworks exist to control AMR, paradoxical trends emerge in the face of agricultural runoff and wastewater management limitations. In the Netherlands, pig farms near agrarian soils have been found to harbor *mcr-1*, a gene conferring resistance to colistin, at concentrations as high as 10^6^ copies per gram of soil. Furthermore, identical plasmids carrying *mcr-1* were detected in human clinical isolates, suggesting that agricultural runoff contributes to transmitting resistant pathogens from the environment to human populations [120]. Similarly, WWTPs have been identified as critical sources of environmental antibiotic resistance in the United States. Although designed to reduce microbial contaminants, these facilities often cannot eliminate clinically relevant resistance genes such as *bla_CTX-M_*, which are associated with resistance to third-generation cephalosporins. Recent studies indicate that ARGs persist through treatment processes, with effluents discharging substantial quantities of resistance genes into surrounding aquatic environments, including major bodies of water like the Great Lakes. This highlights the limitations of current wastewater management strategies and underscores the complexity of mitigating antimicrobial resistance through regulatory frameworks alone [121]. The main regional drivers, transmission pathways, dominant resistance genes, and associated human health impacts of environmental AMR are summarized in Table 3.

### 4.3. Transboundary AMR Spread

Global trade and airborne transmission are two critical vectors contributing to the spread of AMR on a global scale. The international movement of goods, particularly food products, plays a significant role in disseminating resistant pathogens. In Vietnam, colistin resistance genes, such as *mcr-1* and *mcr-3*, have beenidentified in ESBL-producing *E. coli* isolated from food sold in Ho Chi Minh City, indicating a reservoir of resistance genes within the food chain [123]. These resistant strains pose a risk of transboundary transmission when such food products are exported.

A notable example of this risk is observed in Europe, where *mcr-3*-positive Salmonellastrainswere identified in human infections in Denmark between 2009 and 2017. Genomic analysis revealed that some of these cases were linked to recent travel to Southeast Asia, including Vietnam, suggesting a route of transmission facilitated by international travel and trade. These isolates also carried multiple resistance genes, further amplifying the public health threat [124].

The spread of such resistant pathogens through international trade highlights the importance of monitoring and regulating the movement of goods to mitigate the global transmission of AMR.

In addition to trade, airborne transmission has emerged as another significant pathway for the spread of resistant bacteria. Dust storms, particularly those originating from poultry farms in China, have been identified as a means by which AMR bacteria, such as *Streptococcus* spp. carrying *erm*(F) genes, including the Pacific, are dispersed across vast distances. These storms carry particulate matter laden with resistant pathogens, facilitating their introduction into new environments where they can proliferate and spread further. One study found that airborne ARGs were detected up to 10 km downwind from poultry farms, suggesting that wind patterns can facilitate the spread of resistant bacteria into surrounding environments [125].

## 5. Strategies for Mitigating Environmental AMR: A Multidisciplinary Roadmap

**Figure 8:**
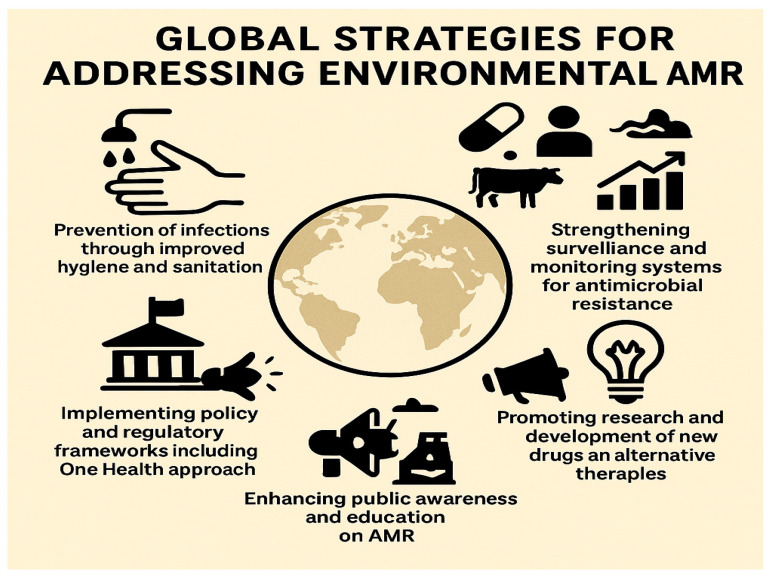
Global strategies for addressing environmental AMR.

The fight against environmental AMR demands coordinated, science-driven strategies that address contamination sources, transmission routes, and global inequities. Below, we synthesize evidence-based interventions, technological innovations, and policy frameworks critical for curbing environmental AMR. The main pillars of these strategies are illustrated in Figure 8.

Schematic representation of key strategies to mitigate environmental antimicrobial resistance: prevention of infections through improved hygiene and sanitation; implementing policy and regulatory frameworks, including the One Health approach; enhancing public awareness and education; promoting research and development of new drugs and alternative therapies; and strengthening surveillance and monitoring systems for antimicrobial resistance.

### 5.1. Regulatory and Policy Interventions

Restricting agricultural antibiotic use has emerged as a critical strategy in addressing AMR in environmental reservoirs, particularly agrarian soils. A notable example of this approach is the European Union’s Veterinary Medicinal Products Regulation (EU 2019/6), which has been instrumental in reducing veterinary antibiotic sales by 43% between 2019 and 2023. This regulation significantly reduced this by banning the prophylactic use of antibiotics in livestock and mandating that veterinary prescriptions be required for antibiotic treatments [126,127]. These regulatory measures are aimed at controlling antibiotic use and minimizing the development and spread of resistance genes in agricultural environments. Similar policies adopted globally, such as the U.S. FDA Guidance #213, have demonstrated the efficacy of such interventions, with studies indicating a 35% reduction in antibiotic resistance gene (ARG) abundance in agricultural soils following these restrictions [128].

Pharmaceutical emission standards are another critical component of AMR mitigation efforts, particularly concerning contaminating water sources with pharmaceutical residues. India’s Zero Liquid Discharge Policy represents a significant regulatory initiative aimed at addressing the environmental impacts of pharmaceutical manufacturing. The policy mandates that drug manufacturers treat effluents to a maximum of <1 μg/L of antibiotic residues, a measure that has proven effective in reducing the presence of the *bla_NDM-1_* gene in local waterways by 60% near Hyderabad [11]. This reduction in antimicrobial contamination is crucial for preventing the environmental dissemination of resistance genes, which can enter the food chain and exacerbate public health threats. In Europe, the EU has implemented a comprehensive surveillance strategy through its Watch List, which monitors the presence of antibiotics such as ciprofloxacin in surface waters. By enforcing strict thresholds of ≤0.1 μg/L, this initiative plays a pivotal role in limiting the environmental spread of pharmaceutical pollutants. These combined efforts to regulate pharmaceutical emissions are essential for reducing the environmental burden of AMR, ensuring that the aquatic microbiomes remain less conducive to the selection and dissemination of resistant strains [129].

### 5.2. Technological Innovations in Wastewater and Manure Treatment

Advanced wastewater treatment technologies play a crucial role in reducing the dissemination of ARGs in the environment. Among these techniques, the combination of ozonation and activated carbon adsorption has proven particularly effective. Studies conducted in Germany have shown that this process can eliminate up to 99.9% of ARGs, including the β-lactamase gene *bla_CTX-M-15_*, with a treatment cost of approximately €0.05 per cubic meter of water. This method has been widely implemented in various wastewater treatment plants, demonstrating its high efficiency and cost-effectiveness [130].

Membrane bioreactors (MBRs) represent another advanced treatment strategy. For instance, the NEWater plant in Singapore uses a 0.1 µm ultrafiltration membrane to achieve a significant reduction in the *mcr-1* gene, which is associated with colistin resistance. MBRs combine biological treatment with physical separation, thereby enhancing water quality and significantly reducing the risk of ARG transmission into the environment [131].

Regarding the treatment of animal manure, thermophilic composting has been shown to be effective. Heating manure to 55 °C for seven days can degrade the *tet(M)* gene—linked to tetracycline resistance—by up to 99%. This high-temperature process accelerates the breakdown of resistance determinants, thereby reducing the likelihood of gene transfer to soil and water ecosystems [132].

Soil amendment with biochar is also emerging as a promising strategy to limit the horizontal transfer of ARGs. Adding 5% (*w*/*w*) of biochar to soil can significantly reduce the availability of extracellular DNA, a key vector in gene transfer between bacteria. This approach not only helps mitigate the spread of ARGs but also enhances soil fertility and promotes carbon sequestration, making it a sustainable option for managing manure and organic waste [133].

Additionally, *gamma-ray irradiation* has been identified as an effective method for mitigating ARB and ARGs in aquatic environments. A study by Zhang et al. (2024) [47] demonstrated that gamma-ray irradiation significantly reduced ARGs, including *tetO*, *tetA*, and *bla_TEM-1_*, in secondary effluents from municipal wastewater treatment plants. The irradiation process led to up to 90.5% removal of these genes and resulted in no resurgence of the ARGs after exposure to gamma rays. This technique not only inactivated ARB but also reduced the extracellular DNA responsible for horizontal gene transfer, thus mitigating the environmental risks associated with antibiotic resistance [47].

### 5.3. One Health Integration

Global coordination efforts to combat AMR have seen significant strides, particularly through initiatives like the WHO-FAO-OIE Tripartite Collaboration. This collaboration launched the Global AMR Surveillance System (GLASS), which has been instrumental in integrating environmental data from over 90 countries, providing a comprehensive global overview of AMR trends and challenges [134]. Such initiatives are crucial in ensuring a coordinated response to AMR, facilitating data sharing, and fostering international cooperation. At a regional level, the European Union’s Joint Programming Initiative on Antimicrobial Resistance (JPIAMR) has also contributed by funding transdisciplinary projects aimed at reducing antibiotic use in agriculture. For example, the DISARM project is one such initiative that focuses on mitigating antibiotic dependence in farming through the adoption of alternative strategies [135].

On the national level, countries like the Netherlands and Thailand have set exemplary models in their efforts to combat AMR. The Netherlands, through its “Farm-to-Fork” model, has achieved a remarkable reduction in veterinary antibiotic usage by 71% between 2009 and 2023. This policy shift has had a significant impact on the prevalence of MRSA in pigs, decreasing it by 90% [136]. This success underscores the effectiveness of integrated approaches to AMR, particularly when coupled with stringent regulations and a commitment to reducing antibiotic use in livestock. Similarly, Thailand’s reforms in the aquaculture sector have led to the replacement of 50% of antibiotics used in shrimp farming with probiotics. This transition has not only improved farm productivity but has also resulted in a 40% reduction in the prevalence of the *qnrS* gene in coastal sediments, a significant marker for fluoroquinolone resistance [137]. These national success stories highlight the potential of well-coordinated, locally tailored interventions in the fight against AMR. Through the implementation of innovative practices and regulations, countries can effectively reduce the environmental burden of resistance, contributing to a global reduction in AMR.

### 5.4. Global Surveillance and Monitoring Networks

Environmental DNA (eDNA) tracking has emerged as a powerful tool in the surveillance of ARGs in various environmental reservoirs, including water, soil, and air. One notable initiative in this field is the MARA project (Monitoring of Antibiotic Resistance in the Environment), which analyzed metagenomic data from over 1400 environmental samples collected across 18 provinces in China between 2013 and 2020. This large-scale study identified and quantified 290 ARGs and 30 mobile genetic elements, including clinically relevant genes such as *bla_NDM_* and *mcr* [138]. By aggregating data from diverse ecological compartments such as surface water, sediment, dust, and soil, the MARA project provides a robust framework for understanding the environmental distribution of resistance genes and identifying potential hotspots of AMR. This effort contributes significantly to global AMR surveillance and supports evidence-based strategies for mitigating the spread of resistant pathogens [139].

In addition to global efforts, regional projects such as ResistoMap have taken eDNA tracking a step further by offering a real-time dashboard that monitors ARG hotspots in European rivers. This system enables continuous tracking of resistance gene dynamics in water bodies, where resistance genes can persist and disseminate, impacting both aquatic ecosystems and human populations. The ResistoMap project provides an important resource for policymakers and environmental health professionals, helping to pinpoint regions with elevated ARG levels, thus facilitating targeted interventions in the management of waterborne resistance. The ability to track ARGs in real time also enables a more rapid response to emerging threats, further enhancing public health preparedness in the face of rising AMR [140].

Furthermore, citizen science initiatives, such as the mBioTracker App, have empowered local communities to contribute to the tracking of ARGs, thus expanding the reach of surveillance efforts. In Kenya, for example, the app allows farmers to upload data on the presence of ARGs in manure, facilitating localized interventions to reduce the spread of resistance within agricultural settings. This collaborative approach not only enhances the spatial and temporal coverage of AMR monitoring but also fosters community involvement in addressing a global health challenge. By leveraging the power of citizen science, the mBioTracker App represents a novel way to integrate grassroots participation into the broader scientific endeavor to combat antimicrobial resistance, especially in regions where resources for traditional monitoring may be limited [141].

### 5.5. Emerging Solutions and Future Directions

Managing and mitigating AMR through innovative strategies is crucial for addressing the growing environmental threats posed by resistant pathogens. One promising approach involves phage-based biocontrol, where engineered bacteriophages have been used to target specific pathogens such as *P. aeruginosa*. In controlled trials, these phages significantly reduced the bacterial population in wastewater systems. A study by Kauppinen et al. (2021) [142] showed that the use of bacteriophages *vB_PaeM_V523* and *vB_PaeM_V524* led to a reduction of more than 2.4 log10 in *P. aeruginosa* populations, highlighting the potential of phages to control resistant strains in environmental reservoirs. This reduction not only underscores the potential of phages as a tool for environmental AMR control but also emphasizes the importance of targeting resistant strains directly in their ecological niches, such as wastewater, where they are prevalent [142].

Another critical intervention in reducing AMR in agricultural settings is vaccine-driven stewardship. For example, the use of poultry vaccines targeting *E. coli* has led to a notable reduction in the reliance on antibiotics in broiler production systems. In a field experiment, the vaccination of broiler chickens with a live attenuated *E. coli* vaccine resulted in improved general health parameters and a significant reduction in the incidence of *E. coli* infections, leading to less antibiotic treatment. This reduction in antibiotic consumption directly impacted the environmental spread of resistance, particularly in agricultural runoff, where the prevalence of resistant *E. coli* strains, including those with the *tet(A)* gene, was lower than in unvaccinated flocks. Such vaccines represent an effective strategy to protect animal health and reduce the ecological burden of AMR, highlighting the role of preventive measures in managing AMR across ecosystems [143].

In addition to biological interventions, technological advancements such as artificial intelligence (AI) are increasingly vital in predicting and managing AMR. Machine learning models, such as DeepARG, have shown exceptional promise in forecasting the emergence of ARGs in environmental soils. With an impressive accuracy rate of 92%, these AI-powered systems enable researchers and policymakers to anticipate potential hotspots of ARG accumulation, allowing for more targeted interventions and more effective stewardship programs. The integration of AI into AMR management strategies offers a new frontier in predictive epidemiology, where data-driven approaches can guide proactive environmental health measures, ultimately reducing the spread of resistance genes across various ecosystems [144].

## 6. Conclusions

Environmental AMR represents an escalating global crisis, driven mainly by anthropogenic pollution. Water, soil, and air have become interconnected reservoirs for ARGs and ARBs, contributing to the widespread dissemination of resistance across ecosystems. The primary forces behind this phenomenon include pharmaceutical waste, agricultural runoff, and inadequate sanitation systems, all of which create selective pressures that accelerate the evolution of resistance in microbial populations. These pressures are particularly potent in environments contaminated by the overuse and misuse of antibiotics and the lack of proper waste management and water treatment infrastructure. In high-income regions, environmental AMR is exacerbated by legacy pollution and intensive agricultural practices, which continue to contribute to the accumulation of antibiotic residues in the environment. In contrast, low-resource settings are often disproportionately affected by unregulated antibiotic use in human healthcare and animal husbandry, combined with poor infrastructure that hampers adequate sanitation and waste treatment efforts.

The environmental transmission of AMR necessitates immediate and coordinated global efforts to mitigate its spread and impact. Addressing this challenge requires robust policy enforcement at the international level, particularly by implementing regulations that limit antibiotic use in agriculture. For instance, the European Union has successfully reduced veterinary antibiotic use by 71%, setting an important precedent for global efforts to regulate antimicrobial consumption in farming. Alongside these regulatory measures, pharmaceutical emission standards must be established to minimize the release of antibiotic residues into the environment, particularly through industrial processes and waste disposal practices. Integrating innovative technologies also plays a critical role in combating environmental AMR. Advanced wastewater treatment techniques, such as ozonation and the use of biochar, can significantly reduce the levels of ARGs and ARBs in water systems. Real-time surveillance systems for tracking ARGs are also vital tools for monitoring resistance trends and enabling rapid response to emerging threats. Furthermore, addressing environmental AMR demands a “One Health” approach that bridges human, animal, and environmental health. Initiatives like the World Health Organization’s Global Antimicrobial Resistance Surveillance System (GLASS) network exemplify this integrated approach, which fosters collaboration across sectors and enhances the global capacity to combat AMR in all its environmental reservoirs. These collective efforts are essential to mitigating the planetary threat AMR poses and ensuring antibiotics’ continued effectiveness in treating infectious diseases worldwide.

## Figures and Tables

**Table 1:** Summary of water and wastewater treatment technologies, their advantages, limitations, and potential contribution to AMR.

Treatment Technology	Main Advantages	Main Limitations	Potential Contribution to AMR	Reference
Conventional Activated Sludge	Cost-effective, widely used	Incomplete removal of antibiotics and ARGs	Residual antibiotics and ARGs in effluent	[41,42]
Membrane Filtration (MBR)	High removal efficiency for bacteria/particles	High cost, membrane fouling	Reduces ARB/ARGs, but not all; possible biofilm formation	[41,42]
Ozonation/Advanced Oxidation	Effective for many compounds	High energy cost, byproducts	Degrades some antibiotics/ARGs, but not all	[42,43]
UV Disinfection	Effective for bacteria, no chemical residues	Ineffective for extracellular DNA; limited by water turbidity	May not remove free ARGs; possible regrowth	[44]
Chlorination	Broad-spectrum disinfection, low cost	Can select for chlorine-resistant bacteria; toxic byproducts	Selection of resistant strains; incomplete ARG removal	[45]
Composting/Biosolids Recycling	Waste valorization, nutrient recycling	ARGs can persist in biosolids; risk if not fully sanitized	Spreads ARGs if not properly treated	[46]
Thermophilic Composting	High temperature degrades many ARGs	Not all ARGs eliminated; energy-intensive	Reduces but does not eliminate resistance genes	[46]
Biochar Amendment	Reduces extracellular DNA availability	Effectiveness varies with soil type and conditions	Limits horizontal transfer of ARGs	[46]
Gamma-ray Irradiation	Inactivates ARB and ARGs efficiently	High cost, limited large-scale application	Reduces ARGs and ARB in treated effluents	[47]

**Table 2:** Key environmental pollutants contributing to AMR (Global Data).

Pollutant Type	Major Sources	Key Resistance Elements	Typical Concentrations	Locations Detected	References
Pharmaceuticals	Drug manufacturing, hospitals	*blaCTX-M*, *qnrS*, *mcr-1*	10–31 mg/L (India), 1.2 mg/L (Europe), 2.5 mg/L (China)	Rivers and lakes in India, Europe, China, Africa, US	[24,32,103]
Wastewater (Treated and Untreated)	Urban wastewater, hospitals	*vanA*, *blaKPC*, *intI1*, ESBLs	Up to 10^8^ ARG copies/mL	Coastal and inland waters, global	[10,11]
Agricultural Runoff	Manure, fertilizers, heavy metals	*ermB*, *tetM*, *sul1*, *czcA*	Up to 10× higher than organic soils	Europe, US, China, Africa	[55,88,104]
Industrial Waste and Microplastics	Textiles, plastics, dyes	*sul1*, *ermB*, *marA*, *blaTEM*	Notably high in estuaries, rivers	UK, China, Europe, Africa	[52,89,105]
Air Pollution	PM2.5, PM10, dust storms	*blaCTX-M*, *mcr-1*, *sul1*	Up to 10^7^ ARG copies/m^2^/day	China, Europe, US, Africa	[19,98]

**Table 3:** Regional drivers and pathways of environmental AMR.

Region	Key Drivers	Primary Pathways	Dominant ARB/ARGs	Human Health Impact	References
Sub-Saharan Africa	Untreated sewage	Water contamination	*blaCTX-M-15*, *mcr-1*	Rise in pediatric MDR diarrhea	[116]
India and SE Asia	Pharmaceutical effluents, intensive aquaculture	River systems, aquaculture	*blaNDM-1*, *qnrS*	High rates of untreatable UTIs, aquaculture-associated resistance	[24,119]
Europe/N. America	Intensive farming, WWTP effluents	Agricultural runoff, WWTP effluents	*erm(B)*, *tet(M)*, *blaCTX-M*	MRSA in farmworkers; environmental dissemination of ESBLs	[120,121]
Latin America	Urban slums	Urban waterways	*blaOXA-48*, *blaKPC*, *blaVIM-2*, *qnrS*	Hospital CRE outbreaks, community transmission	[122]

## Data Availability

The data presented in this study are available within the article. Raw data supporting this study are available from the corresponding author upon reasonable request.
